# Optimum Water Quality Monitoring Network Design for Bidirectional River Systems

**DOI:** 10.3390/ijerph15020195

**Published:** 2018-01-24

**Authors:** Xiaohui Zhu, Yong Yue, Prudence W. H. Wong, Yixin Zhang, Jianhong Tan

**Affiliations:** 1 Department of Computer Science and Software Engineering, Xi’an Jiaotong-Liverpool University, Suzhou 215123, China; xiaohui.zhu@xjtlu.edu.cn; 2 Department of Computer Science, University of Liverpool, Liverpool L69 3BX, UK; pwong@liverpool.ac.uk; 3 Department of Computer Science and Software Engineering, Nantong University, Nantong 226019, China; 4 XJTLU-Huai’an Research Institute of New-Type Urbanization, Huai’an 223005, China; yixin.zhang@xjtlu.edu.cn; 5 Jiangsu Province Hydrology and Water Resources Investigation Bureau Suzhou Branch, Suzhou 215011, China; Jianhong_tan@hotmail.com

**Keywords:** multi-objective particle swarm optimization, water quality monitoring network, optimum monitoring network design, bidirectional water flows, storm water management model

## Abstract

Affected by regular tides, bidirectional water flows play a crucial role in surface river systems. Using optimization theory to design a water quality monitoring network can reduce the redundant monitoring nodes as well as save the costs for building and running a monitoring network. A novel algorithm is proposed to design an optimum water quality monitoring network for tidal rivers with bidirectional water flows. Two optimization objectives of minimum pollution detection time and maximum pollution detection probability are used in our optimization algorithm. We modify the Multi-Objective Particle Swarm Optimization (MOPSO) algorithm and develop new fitness functions to calculate pollution detection time and pollution detection probability in a discrete manner. In addition, the Storm Water Management Model (SWMM) is used to simulate hydraulic characteristics and pollution events based on a hypothetical river system studied in the literature. Experimental results show that our algorithm can obtain a better Pareto frontier. The influence of bidirectional water flows to the network design is also identified, which has not been studied in the literature. Besides that, we also find that the probability of bidirectional water flows has no effect on the optimum monitoring network design but slightly changes the mean pollution detection time.

## 1. Introduction

River systems play a crucial role in the sustainable development of a community. However, overexploitation and increasing pollution of this vital resource are threatening our ecosystems and even the life of future generations. On the one hand we need more and more clean water, and on the other hand, industry and living activities create more and more pollutants in freshwater sources. Water quality monitoring has become one of the routine efforts for environmental protection all over the world. However, designing water quality monitoring networks is a very complex process due to the large number of factors to be considered such as monitoring locations, selection of water quality parameters, monitoring frequency, and identification of monitoring objectives [1]. The problem of planning and optimizing water quality monitoring programs (WQMPS) has been widely studied since the 1940s [2,3,4,5,6,7,8,9].

With the rapid development of computer science and communication and sensor technologies, water quality parameters from more locations can be remotely detected and transmitted by automatic monitoring stations, resulting in a much higher monitoring frequency, more monitoring data, and better monitoring efficiency. However, the costs of building and operating an automatic monitoring station are very high. The successful water quality monitoring relies on the availability of low-cost and high efficient monitoring network to collect appropriate and reliable data [10]. Optimization design of the water quality monitoring network can help us to build a cost-effective monitoring network and improve the monitoring performance and reduce the construction and operating costs, which is essential for the sustainable development of water quality monitoring networks. Many researchers have studied the optimum design of water quality monitoring network for river systems based on a variety of optimization objectives and approaches. Quyuan et al. (2008) used a single objective generic algorithm (GA) to design an optimum monitoring network based on a geometric analysis and a simple application in a hypothetical river system. The spatial distribution of the monitoring stations and the expected number of monitoring stations required to locate the pollutant source are associated together as one optimization objective in this algorithm. However, practical river systems are complex and other factors such as flow rate and river depth and width should also be considered while designing the monitoring network. Telci et al. (2008) argued that the design of an optimal water quality monitoring network should mainly focus on two objectives—minimal pollution detection time and maximal detection reliability—and calculated the optimal placement of monitoring devices using the GA under relatively simple discrete spatial distributions on spill events. They also applied this methodology to the Altamaha river basin to identify the locations of the best monitoring stations in the river system [11]. However, the Pareto frontier of the optimization results was not mentioned in this paper, resulting in difficulties for evaluating all the optimization results. Park (2013) used stochastic discrete optimization via a simulation (QvS) algorithm and a penalty function with memory (PFM) to the optimal location of a finite number of monitoring positions that minimize the expected detection time of a contaminant spill event while guarantee a higher detection probability [12]. However, the penalty value significantly increases the detection time of a deployment solution when the detection probability is less than 100%. Chang and Lin (2014) selected seven criteria to evaluate the suitability of the water quality monitoring design and used fuzzy theory to improve the objectivity in the data classification and ranking [4]. However, it is very difficult to collect detailed information (e.g., percentage of farmland and built-up area and green cover ratio) to satisfy all the criteria of the algorithm. Changhyoun Lee et al. (2014) used Shannon entropy to simplify the optimization procedure [6]. Mahyar Aboutalebi et al. (2016) proposed a multi-objective design of water quality monitoring networks using CE-QUAL-W2 and NSGAII-SVR methods [13].

Most of the research simplify the model of river systems and only unidirectional water flow is considered to design an optimum water quality monitoring network. However, affected by regular tides, some river systems have bidirectional water flows. For example, Taihu Lake Basin is located in the east of China with an area of 36,900 km^2^. There is about 120,000 km length of river networks in Taihu Lake Basin. Affected by tides, some river networks have bidirectional water flows (e.g., Wangyu River, Xinmeng River, and Liu River). To the best of our knowledge, the effect of bidirectional water flows has not been studied, and we do not know how far the bidirectional water flows can affect the optimum design of water quality monitoring network. In this study, we emphasize the dynamic behavior of water flow directions affected by regular tides and pollution transports along the river system.

## 2. Methodology

### 2.1. Hydraulic Simulations

The Storm Water Management Model (SWMM) is a dynamic rainfall-runoff simulation model used for single event or long-term (continuous) simulation of runoff quantity and quality from primarily urban areas. It can track the quantity and quality of runoff generated within each sub-catchment, and the flow rate, flow depth, and quality of water in each pipe or channel during a simulation period [14]. It also can be used to simulate the flow and water quality in open-channels, i.e., in river systems. Here, we use SWMM to simulate the pollution events and pollutant transport along the river system. To compare our study results with the achievements given by [11,15,16] based on the same hydraulic processes and parameters, we also use SWMM and the same hypothetical river network used in the literature.

**Figure 1:**
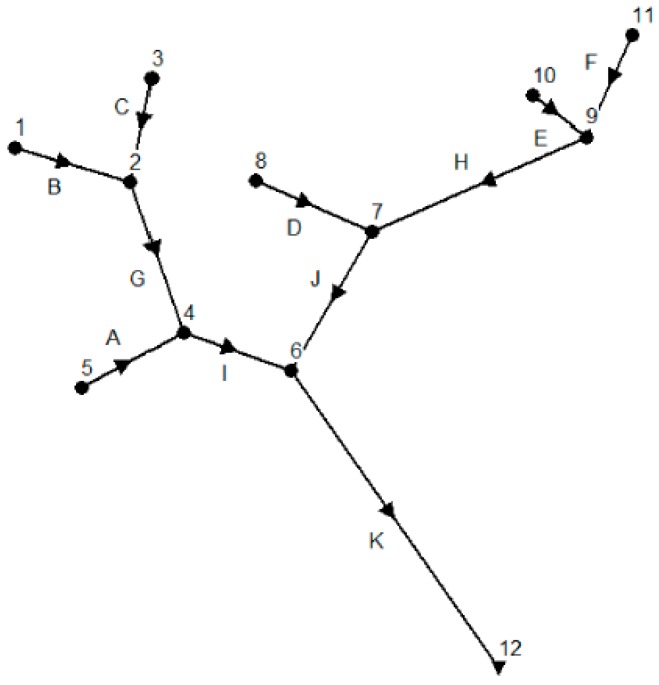
Hypothetical river network A.

We build a river network using SWMM shown in Figure 1. The hypothetical river network A has six inlet nodes (nodes 1, 3, 5, 8, 10, and 11), five intermediate nodes (nodes 2, 4, 6, 7, and 9), and one outlet node (node 12). We assume that a pollution event can occur at any node randomly with the same amount of pollutant spilling and that there is only one pollution event at each time. In order to get steady water flows when a pollution event occurs, we simulate the water flows for 24 h from 00:00 to 23:59 with a steady water flow of 283.17 L/s (10 ft^3^/s) for each inlet node and the pollution event occurs at 10:00 and lasts for 1 h. We also assume that the pollutant concentration is 10 mg/L when a pollution event occurs at inlet nodes, resulting in 10.19 kg of the total amount of pollutant spilling for each pollution event. In a real water monitoring environment, monitoring sensors and devices can hardly distinguish whether a pollutant is conservative or no-conservative. So, we assume that the pollutant is conservative in our paper. To obtain the same pollution level during simulations, we also set the same amount of pollutant spilling of 10.19 kg for intermediate nodes 2, 4, 6, 7, 9, and outlet node 12 when a pollution event occurs at these nodes. The remaining characteristics of the river network A is shown in Table 1, which is the same used by Telci.

### 2.2. Optimum Objectives

The purpose of designing an optimum water quality monitoring network is, given a river system being monitored and a definite number of monitoring devices according to the budget available for constructing, to try to find an optimum deployment solution to maximize the pollution detection probability and minimize the pollution detection time within all the potential monitoring locations. In this study, we consider two optimization objectives of minimum pollution detection time and maximum pollution detection probability, which are the same as those in Telci’s paper. In addition, we also emphasize the dynamic behavior of pollution transports along the river system and water flow directions affected by tides.

#### 2.2.1. Minimum Pollution Detection Time

Assume that we deploy *n* monitoring devices in a river system out of *m* potential monitoring locations (*n* ≤ *m*), which means *n* special monitoring locations will be selected to deploy monitoring devices from *m* potential monitoring locations. It is easy to know that the total number of potential deployment solutions *T* is

(1) $$T=C_{m}^{n}$$

where *m* is the number of potential monitoring locations and *n* is the number of monitoring devices deployed in a river system. For a given optimum deployment solution *S_k_* = [*s_k_*_1_, *s_k_*_2_, *s_ki_*, …, *s_kn_*], where *s_ki_* is the index of selected monitoring locations, *k* ≤ *T* and *s_ki_* ≤ *m*. Let *d_i_^j^*(*S_k_*) be the pollution detection time of monitoring location *i* when a pollution event occurs at location *j*. The minimum pollution detection time for location *j* is

(2) $$d^{j}\left(S_{k}\right)=\min\left\{d_{1}^{j}\left(S_{k}\right),d_{2}^{j}\left(S_{k}\right),\;\ldots ,\;d_{n}^{j}\left(S_{k}\right)\;\right\}$$

where *j ≤ m*. For a definite optimum deployment solution *S*, the set of minimal pollution detection time for all potential locations is *d*(*S_k_*) = [*d*^1^(*S_k_*), *d*^2^(*S_k_*), …, *d^j^*(*S_k_*) …, *d^m^*(*S_k_*)]. Assume $\bar{d\left(S_{k}\right)}$ is the mean value of all minimum pollution detection time at all *m* potential monitoring locations for the given solution *S_k_*, $\bar{d\left(S_{k}\right)}$ is

(3) $$\bar{d\left(S_{k}\right)}=\frac{1}{m}\overset{m}{\underset{j=1}{\sum }}d^{j}\left(S_{k}\right)$$

Let $\bar{d\left(S\right)}$ be the minimum mean pollution detection time for all potential deployment solutions, we can get the following equation

(4) $$\bar{d\left(S\right)}=\min\left\{\bar{d\left(S_{1}\right)},\bar{d\left(S_{2}\right)},\;\ldots ,\bar{d\left(S_{T}\right)}\;\right\}$$

where *T* is the total number of deployment solutions shown in Equation (1). One of our two objectives is to find a deployment solution which has the minimum mean pollution detection time, as shown in Equation (4).

#### 2.2.2. Minimum Pollution Detection Probability

Let *R*(*S_k_*) be the ratio of successful pollution detection scenarios to all potential detection scenarios for a given deployment solution *S_k_*. We get *R*(*S_k_*) as

(5) $$R\left(S_{k}\right)=\frac{1}{m}\overset{m}{\underset{i=1}{\sum }}r_{i}$$

where *k* ≤ *T*, *m* is the amount of potential monitoring locations. *r_i_* = 1 if the pollution event at location *i* can be detected by the deployment solution *S_k_* or *r_i_* = 0 if the pollution event cannot be detected. Let *R*(*S*) be the maximum pollution detection probability within all the potential deployment solutions.

(6) $$R\left(S\right)=\max\left\{R\left(S_{1}\right),R\left(S_{2}\right),\ldots ,R\left(S_{T}\right)\right\}$$

where *T* is the total number of potential deployment solutions. Our second objective is to find a proper deployment solution which has a maximum pollution detection probability as Equation (6) shows.

### 2.3. MOPSO Algorithm

On the one hand, we can find from Equation (1) that when we increase the value of *m* and/or *n*, the number of potential deployment solutions will be increased exponentially. For example, if we deploy 20 monitoring devices out of 100 potential locations, the number of the deployment combinations is about 10^30^, which is too large to obtain the optimum deployment results using enumeration search methods within a reasonable time. On the other hand, these two optimum objectives normally conflict with each other, which means that we aim to find some good trade-off solutions among these objectives [17]. So, some optimization methodologies should be used here to save the computing time and converge to optimum results in a reasonable period of time.

MOPSO is one of the more popular evolution algorithms used in recent years [18]. The Pareto dominance is used in MOPSO to handle multi-objective functions and improve the PSO algorithm to be able to deal with multi-objective optimization problems [19]. The algorithm uses a secondary repository of particles that is later used by other particles to guide their own flight and the special mutation operator to enrich the exploratory capability. In order to know how competitive MOPSO was, Coello et al. (2004) compared it against three state-of-the-art multi-objective evolutionary algorithms of Nondominated Sorting Genetic Algorithm II (NSGA-II), Pareto Archived Evolution Strategy (PAES), and Microgenetic Algorithm for Multi-Objective Optimization (MicroGA) using five different test functions. Experimental results show that MOPSO has a highly competitive performance and can be considered a viable alternative to solve multi-objective optimization problems, and it can cover the full Pareto frontier of all the potential solutions with low computational time. Here, we use MOPSO to calculate Pareto frontier for the optimal water quality monitoring network design and compare the results to the literature. The velocity and position of particles during the computing iteration are updated by the following equations:

(7) $$V_{i}\left(t+1\right)=\omega V_{i}\left(t\right)+c_{1}r_{1}\left(pbest\left(i,t\right)-p_{i}\left(t\right)\right)+c_{2}r_{2}\left(gbest\left(i,t\right)-p_{i}\left(t\right)\right)$$

(8) $$p_{i}\left(t+1\right)=p_{i}\left(t\right)+V_{i}\left(t+1\right)$$

where *V* denotes the particle’s velocity, *ω* is an inertia weight constant, *r*_1_ and *r*_2_ are uniformly distributed random variables within range (0, 1), *pbest*(*i*,*t*) is the best position that particle *i* has had, *gbest*(*t*) is the best position in all current particles, and *c*_1_ and *c*_2_ are positive constant coefficients for acceleration. The pseudocode of MOPSO is shown in Algorithm 1.

The classical MOPSO is a powerful algorithm used to find global optimum results for continuous definition domains. However, it cannot be applied to discrete problems directly. Here, we define a new fitness function to calculate the cost of each particle using a round function to map the continuous value of a particle to a discrete space, which represents the number of potential monitoring locations. Algorithm 2 shows the pseudocode for the fitness function. Assume that we deploy *n* monitoring devices in the hypothetical river system shown in Figure 1. Each particle is composed of *n* different values, and each value represents a monitoring location. The main idea of the fitness function is as follows. First, we decompose the particle into *n* separate real values and then get *n* integers using a round function. The *n* integers represent the number of *n* potential monitoring locations respectively. Second, we search each row in pollution detection time table obtained from the pollution simulation by SWMM (e.g., Table 2) and calculate the minimum detection time for each potential pollution event. Finally, we calculate the mean detection time and the detection probability for this particle.

As we mentioned above, we try to find optimum monitoring deployment solutions with minimum mean pollution detection time and maximum detection probability. However, MOPSO always requires minimal parameter values to calculate the Pareto frontier. So, we calculate the mean pollution detection time and the reciprocal of pollution detection probability in our fitness function to satisfy this special requirement of MOPSO. In our fitness function, if a pollution event cannot be detected in a deployment scenario (*detectTime* = ’-’), we will not count it in the mean pollution detection time but will calculate it in the pollution detection probability. This is different from Telci’s paper. They used a penalty value for non-detection scenario, which significantly increases the final pollution detection time when the pollution detection probability is less than 100%.
**Algorithm 1** Pseudocode of MOPSO Algorithm**Procedure MOPSO****Step 1.** Algorithm initialization  (1) Initialize all parameters   (*e.g., size of population and repository, maximum number of iterations, search space*)  (2) For each particle do    (a) Initialize the particle‘s position randomly    (b) Initialize *pbest* with its initial position    (c) Initialize particle’s velocity *V_i_* = 0  (3) Calculate non-domination particles using fitness function  (4) Initialize *gbest* with a particle selected from non-domination particles using a roulette wheel selection**Step 2.** Repeat until the termination criteria is met or to the maximum number of iterations  (5) For each particle do    (a) Calculate particle’s new velocity using Equation (7)    (b) Calculate particle’s new position using Equation (8)    (c) Update particle’s *pbest*    (d) Calculate non-domination particles using fitness function    (e) *gbest* = a particle selected from non-domination particles using a roulette wheel selection**Step 3.** Output non-domination particles**End Procedure**
**Algorithm 2** Pseudocode of Fitness Cost Function**Procedure Fitnesscost (*Particle p*)**Array *pos* ← [*n* position values in particle *p*] Array *loc* ← [ ]For each *element* in *pos* do   *node* ← round(*element*)  *loc* ← *node*end for*meanTime* ← *0**count* ← *0**probability* ← *0*for each *row* in *Table 2* do   *detectTime* ← *MAX*
  for each *l* in *loc* do    *detectTime* ← min (*detectTime*, *row(l)*)  end for  if *detectTime* ≠ *MAX* then    *meanTime* ← *meanTime* + *detectTime*
    *count* ← *count* + 1  end ifend for*meanTime* ← *meantime*/*count*
*probability* ← *row.length*/*count*Return (*meanTime*, *probability*)**End Procedure**

## 3. Simulations and Analysis

In practical water quality monitoring applications, the number of monitoring stations is mainly restricted by several factors such as the total costs of building and operating the infrastructure, the requirement of monitoring performance, etc. In order to gain a deeper understanding about how the dynamic characteristics of a river system affect the optimum design of water quality monitoring network, we carry out four groups of simulations in the following section. We also assume that only 3 monitoring devices will be deployed within the 12 potential monitoring locations.

### 3.1. Simulation for River Network A with a Pollution Detection Threshold of 0.01 mg/L

Before simulation, we set the simulation options for the hypothetical river network A shown in Figure 1. We use the Kinematic Wave routing model and the Horton infiltration model in the simulation. We let the reporting time step and routing time step be 60 s and 30 s separately. Simulation results show that the continuity error for flow routing and quality routing are only −0.77% and 0.00% respectively. We simulate pollution events at each potential monitoring location and get pollution time and pollutant concentration from the report generated by SWMM. A simple program is also developed to automatically calculate the pollution detection time for each potential monitoring location according to the pollutant detection threshold.

Table 2 shows the simulation results of pollution detection time for each potential monitoring location when we set the pollution detection threshold to 0.01 mg/L. The value of ‘-’ in Table 2 represents an infinite value, which means the pollution event cannot be successfully detected at a monitoring location. For example, the first row in Table 2 demonstrates a scenario that a pollution event occurs at location 1 and can be detected at locations 1, 2, 4, 6, and 12. The pollution detection time for these locations are 0 (detected immediately), 27, 81, 118, and 198 min respectively. However, this pollution event cannot be detected at locations 3, 5, 7, 8, 9, 10, or 11 because the polluted water flow cannot reach these locations.

**Figure 2:**
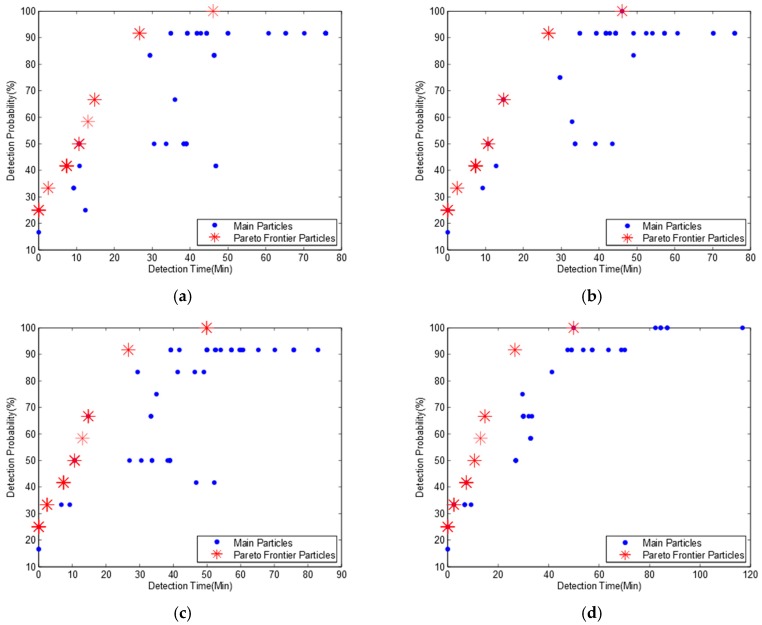
Pareto frontiers for river network A with three monitoring nodes and a detection threshold of 0.01 mg/L. (**a**) Pareto frontier of the first simulation; (**b**) Pareto frontier of the second simulation; (**c**) Pareto frontier of the third simulation; (**d**) Pareto frontier of the fourth simulation.

We run the MOPSO algorithm based on data in Table 2. For the validation of MOPSO to confirm whether the simulation results are steady or not, we run the simulation several times. The simulation results show that though the main particles are quite different from each other, their Pareto frontiers are almost the same. Figure 2 shows four Pareto frontiers in four different sub-diagrams with eight non-dominated particles. The mean pollution detection time, pollution detection probability and optimum monitoring locations for each non-dominated particle are shown in Table 3.

Table 3 indicates that if we deploy three monitoring devices at locations 6, 9, and 12 respectively, all the pollution events can be detected, which is the same as the result in Telci’s paper. If monitoring devices are deployed at locations 2, 6, and 9, the pollution detection probability will be slightly decreased to 91.7% while the mean pollution detection time is also reduced from 45.8 min to 26.6 min. It is also the second maximum pollution detection probability on the Pareto frontier. However, the second maximum pollution detection probability in Telci’s paper is 83%, and the monitoring locations are 4, 7, and 9, which can also be found in our main particles in Figure 2, but it is not a non-dominated particle. Based on this observation, we confirm that our algorithm can get a better Pareto frontier and more detailed optimal deployment solutions. It should be noted that some deployment solutions in Table 3 have much lower pollution detection time and pollution detection probability than others. Though these deployment solutions are also from non-dominated particles, they have little chance to be selected from an engineering point of view.

Telci et al. (2008) used a penalty for non-detected pollution scenarios resulting in a much higher pollution detection time for non-100% detected pollution scenarios. We argue that it is not reasonable, because the mean detection time represents how long the pollution event will be detected if it can be detected by current monitoring network. On the contrary, if a pollution event cannot be detected, the detection probability will be decreased to reflect this non-detected scenario. So, we ignore these non-detected pollution events when we calculate the mean pollution detection time, which results in a shorter mean pollution detection time than in Telci’s paper.

Comparing Table 3 to Figure 2, we find that there are 13 different monitoring deployment solutions mapping to eight non-dominated particles. This is because some deployment solutions with different monitoring locations have the same mean detection time and detection probability, and they map to a same non-dominated particle.

**Figure 3:**
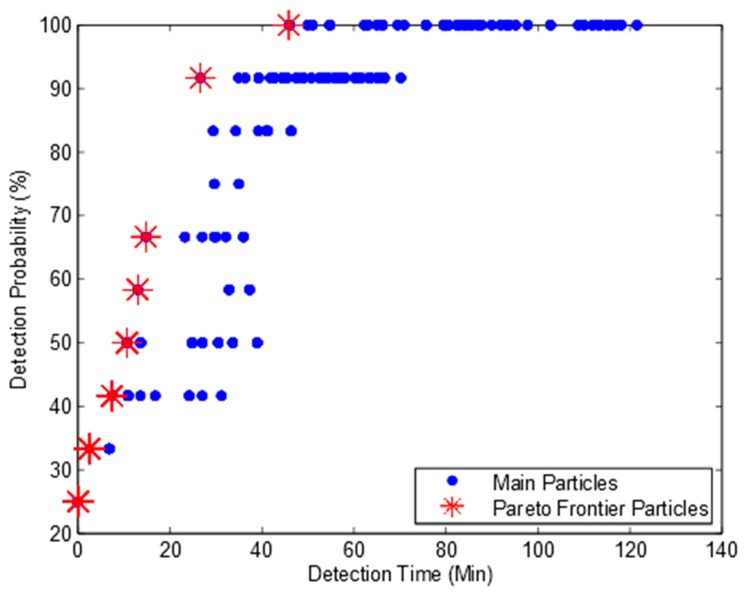
Pareto frontier of enumeration search.

To further confirm whether our algorithm can obtain a full Pareto frontier or not, we developed an enumeration search algorithm. It can exhaustively search all the combinations of potential deployment solutions and obtain all non-dominated deployment solutions. Figure 3 shows the Pareto frontier. We can find from Figure 2 and Figure 3 that the enumeration search algorithm obtains much more particles than our algorithm. This is because the enumeration search algorithm lists all the possible combinations. However, both our algorithm and enumeration search algorithm obtain the same Pareto frontier with eight Pareto particles. Based on this observation, we can confirm that our algorithm can obtain the full Pareto frontier and is suitable to be used for the optimum design of water quality monitoring network.

### 3.2. Simulation for a Reversed River Network B with Pollution Detection Threshold of 0.01 mg/L

**Figure 4:**
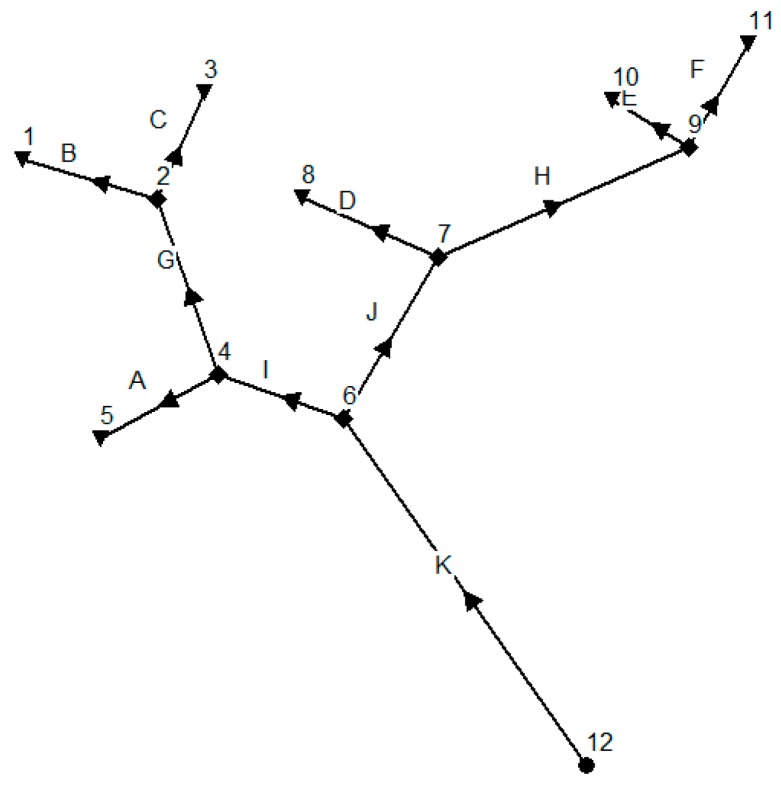
Hypothetical river network B.

Most of the literature only considers the unidirectional water flow. However, influenced by tides, some river systems have bidirectional water flows. In order to observe how far the bidirectional water flows can affect the monitoring network optimization, we create river network B shown in Figure 4 with the same parameters and settings as river network A in Figure 1 but having a reversed water flow direction, resulting in a new river network with six outlet nodes, five intermediate nodes, and only one inlet node. We set the water flow rate of inlet node 12 to 60 ft^3^/s, which is as same as the water flow rate at outlet node 12 in Figure 1. We run the hydraulic simulation in SWMM again and obtain pollution detection time shown in Table 4. We can find from Table 2 and Table 4 that when we reverse the water flow, we get a transposed pollution detection time matrix.

**Figure 5:**
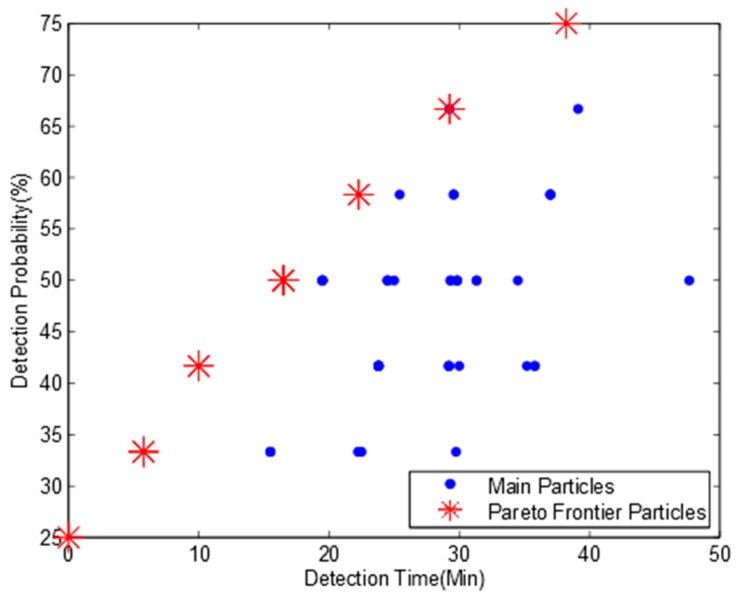
Pareto frontier for river network B with three monitoring nodes and a detection threshold of 0.01 mg/L.

Due to the page limit, only one MOPSO Pareto frontier is shown here in Figure 5. The optimum deployment solutions are shown in Table 5.

We find that when we reverse the water flow direction, there are seven non-dominated particles in Pareto frontier and there is no 100% detection probability solution for river network B. The maximum pollution detection probability is decreased to 75% with a mean pollution detection time of 38.2 min and the optimization monitoring locations are 3, 5, and 10. This is because there are six outlet locations in river network B, and only three monitoring devices cannot detect all the pollution scenarios occurred randomly in 12 potential locations.

Comparing Table 5 to Table 3, we find that the optimization results for both water flow directions are quite different. Based on this observation, we argue that the water flow direction has a significant effect on optimization results of monitoring network design even for the same river system and we should consider the bidirectional water flows when we design an optimization monitoring network for a river system affected by tides regularly.

### 3.3. Simulation with Bidirectional Water Flows

For having a deep insight of the influence of bidirectional water flows for an optimum monitoring network design, we calculate the mean pollution detection time for both the original river network A (Figure 1) and the reversed river network B (Figure 4) at the same time based on the data of pollution detection time in Table 2 and Table 4. As water flow directions can be changed regularly due to tides and the duration for each flow direction may not be equal in a river system. So, we consider two scenarios here when a pollution event occurs:

- Both water flows have the same probability in a river system;
- The probability of two water flows are different.

We slightly modify the previous fitness function in Algorithm 2 and add two extra parameters of *probA* and *probB* in the procedure to denote the probability of the two water flows in a river system. We calculate the pollution detection time and pollution detection probability for bidirectional water flows respectively and get the final mean pollution detection time and probability for two water flows at last. The new fitness function is shown in Algorithm 3.

#### 3.3.1. Bidirectional Water Flows with the Same Probability

**Figure 6:**
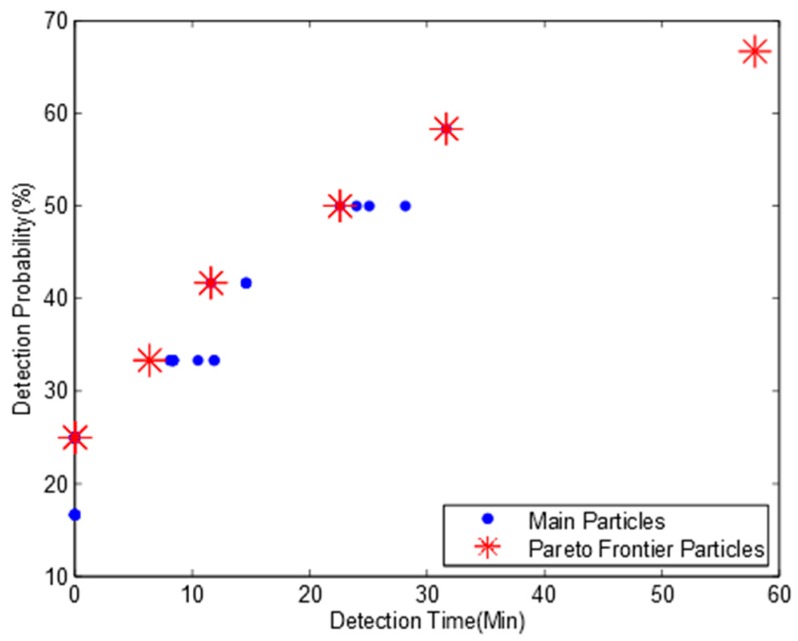
Pareto frontier for bidirectional water flows with three monitoring nodes and a detection threshold of 0.01 mg/L.

We let *probA* and *probB* in Algorithm 3 be 0.5 separately to assume that each water flow with a different direction has the same probability. The simulation results of Pareto frontier and optimization monitoring locations are shown in Figure 6 and Table 6.

Comparing Table 6 to Table 3 and Table 5, we observe that when we consider the bidirectional water flows, the maximum detection probability is decreased from 100% (in Table 3) and 75% (in Table 5) to 66.7%, respectively, while the mean pollution detection time is increased from 45.8 and 38.2 min to 57.9 min. This is because we consider the pollution detection time and detection probability for each water flow respectively and combine them together to obtain the mean pollution detection time and probability based on the time ratio of two reversed water flows, which will significantly increase the pollution detection time and decrease the detection probability. We also find that the optimum deployment solutions are quite different from the previous results in Table 3 and Table 5 and the deployment solution of monitoring locations 3, 10, 12 has the highest pollution detection probability of 66.7% with a mean pollution detection time of 57.9 min.
**Algorithm 3** Pseudocode of Bidirectional Fitness Function
**Procedure BidirectionalFitnessCost** (*Particle p*, *probA*, *probB*)Array *pos* = *n* position values in particle *p*Array loc =[]For each *element* in *pos* *node* = round(*element*) *loc* = *node*EndFor*meanTime* = 0*count* = 0*probability* = 0For each *row 1* in *Table 2* and *row 2* in *Table 4* *detectTimeA* = MAX *detectTimeB* = MAX For each *l* in *loc*  *detectTimeA* = min (*detectTimeA*, *row 1(l)*)  *detectTimeB* = min (*detectTimeB, row 2(l)*)   EndFor   If *detectTimeA* ≠ MAX & *detectTimeB* ≠ MAX then    *avgTime = detectTimeA*
*
*porbA + detectTimeB*
*
*probB*    *meanTime* = *meanTime + avgTime*  *count = count + 1*  EndIfEndFor*meanTime* = *meanTime/count**probability* = *row.length/count*Return (*meanTime, probability*)End Procedure

#### 3.3.2. Bidirectional Water Flows with Different Probabilities

**Figure 7:**
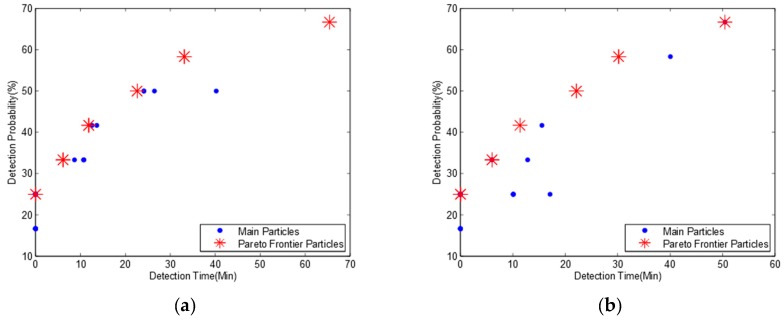
Pareto frontiers for bidirectional water flows with three monitoring nodes and a detection threshold of 0.01 mg/L. (**a**) Probability ratio of river networks A and B is 70:30; (**b**) Probability ratio of river networks A and B is 30:70.

Here we assume that two water flows in a river system have different probabilities. We consider two scenarios: (1) the probability of the water flow as river network A is 70% and the reversed water flow as river network B is 30%. (2) the probability of the water flow as river network A is 30% and the reversed water flow as river network B is 70%. We set the parameter of *probA* to 0.7 and *probB* to 0.3 for the first scenario and exchange the value with each other for the second scenario. We obtain two Pareto frontiers in Figure 7 and two pollution detection time and probabilities in Table 7.

We find from Table 6 and Table 7a, that though we set 70% probability for river network A and 30% probability for river network B, we get the same optimization monitoring locations and detection probabilities while the pollution detection time is slightly increased. This is because the pollution detection time for river network A (Table 3) is slightly higher than for river network B (Table 5) resulting in a higher mean pollution detection time. When we reverse the probability of the two water flows, we get similar results but with a lower mean pollution detection time in Table 7b.

Comparing Table 7a to Table 7b, we observe that though we exchange the probabilities of two water flows, we obtain the same optimal monitoring locations and the same detection probability while the pollution detection time is slightly increased.

Based on the observation above, we draw a conclusion that the bidirectional water flows have a significant effect on an optimal monitoring network design. However, the different probabilities of bidirectional water flows have no effect on the optimization results of monitoring location selection or the pollution detection probability but slightly affect the pollution detection time.

### 3.4. Higher Pollution Detection Threshold for Bidirectional Water Flows

To observe how far the pollution detection threshold can affect the optimum deployment solution for a bidirectional water flow river system, we assume two bidirectional water flows have the same probability and set the pollution detection threshold to 1 mg/L and 2 mg/L respectively. We run the hydraulic simulation in SWMM again based on river networks A and B. Table 8 and Table 9 show four pollution detection time tables for both detection thresholds.

We find that all the pollution detection time in Table 8a are much higher than in Table 2 except for non-detected scenarios. This is because when we increase the pollution detection threshold from 0.01 mg/L to 1 mg/L for river network A, it will take more time to reach a certain pollutant concentration at each potential monitoring location before pollutants can be detected, which will significantly increase the pollution detection time.

Comparing Table 8a to Table 8b, we find that all the pollution events can be successfully detected at location 12 when the pollution detection threshold is 1 mg/L. However, no pollution event can be detected at location 12 when the pollution detection threshold is 2 mg/L, even if the pollution event occurs at location 12 itself. This is because the pollution detection threshold is so high that it is even higher than the maximum pollutant concentration at location 12 when any pollution event occurs.

**Figure 8:**
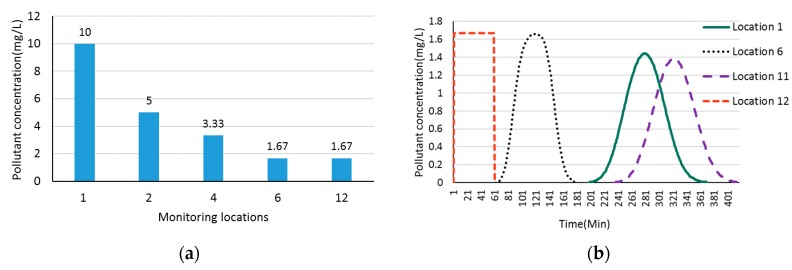
Dilution and changing process of pollutant concentration. (**a**) Dilution process of pollutant concentration when a pollution event occurs at location 1; (**b**) Changing process of pollutant concentration at location 12 when pollution event occurs at locations 1, 6, 11, and 12, respectively.

Figure 8a shows the pollutant dilution along downstream locations when a pollution event occurs at the upstream location 1 in the hypothetical river network A (Figure 1). We can find that the pollutant concentration is decreased from maximal value of 10 mg/L at location 1 to minimal value of 1.67 mg/L at outlet location 12 along the downstream. Figure 8b demonstrates the changing process of pollutant concentration at location 12 when a pollution event occurs at monitoring locations 1, 6, 11, and 12, respectively. We can see from Figure 8b that when a pollution event occurs at location 1, the pollutant will arrive at location 12 in 198 min and will be completely discharged in 368 min with a maximum pollutant concentration of 1.44 mg/L in the pollution event duration. When the pollution occurs at location 12 itself, the pollutant will be diluted by upstream water flows and the maximum pollutant concentration is only 1.67 mg/L. That is why none of the pollution events can be detected when we set pollution detection threshold to 2 mg/L. We get similar results in Table 9 when we increase the pollution detection threshold to 1 mg/L and 2 mg/L, respectively, for river network B.

**Figure 9:**
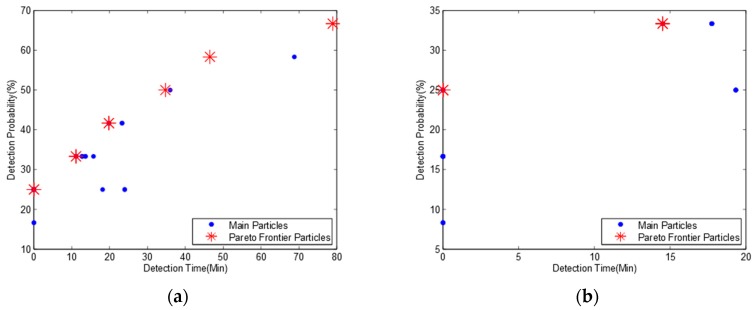
Pareto frontiers for bidirectional water flows with three monitoring nodes and higher detection thresholds. (**a**) Pollution detection threshold = 1 mg/L; (**b**) Pollution detection threshold = 2 mg/L.

Figure 9 shows the Pareto frontier for bidirectional water flows based on the pollution time data in Table 8 and Table 9. We can find that Figure 9a is quite different from Figure 9b, and there are five Pareto frontier particles in Figure 9a but only two Pareto frontier particles in Figure 9b. Table 10 shows the detailed pollution detection time and probability.

Comparing the monitoring location distribution in Table 6 and Table 10a, we observe that though we increase the pollution detection threshold from 0.01 mg/L to 1 mg/L, the two optimum deployment solutions are the same while the detection time is slightly increased. However, from Table 10b, we know that when we continue to increase the pollution detection threshold to 2 mg/L, which is higher than the maximum pollutant concentration in pollution events, the pollution detection probability is significantly decreased, and we get quite different optimum solutions. Based on the observation above, we make a conclusion that a slight change of monitoring device’s pollution detection threshold may not affect the design of optimum monitoring network when the threshold is smaller than the maximal pollutant concentration in the pollution events.

In addition, we consider pollution events with different flow rates to simulate different flow regimes in different seasons. Results indicate that the change of flow rate can affect the optimal deployment solutions. This is because different flow rates result in different pollution detection time at monitoring locations. As we know, the transport processes such as hydrodynamic dispersion and advection can affect the flow rate. So, it also changes the pollution detection time at monitoring locations and ultimately affects the optimal deployment solutions.

## 4. Conclusions

We have presented a novel algorithm based on a modified MOPSO algorithm for the optimum water quality monitoring network design and identification of the influence of bidirectional water flows. We develop new fitness functions for MOPSO to achieve the discrete optimization, which leads to fewer search iterations and can speed up the convergence. Simulation results show that our algorithm can obtain a better Pareto frontier than GA. A bidirectional fitness function is also developed to handle the bidirectional water flows with different probabilities. We find that bidirectional water flows in a river system have a significant effect on the optimum design of water quality monitoring network, and the deployment result is quite different from the same river system with a unidirectional water flow. However, the probability of bidirectional water flows in a river system has no effect on the optimum monitoring network design but will slightly affect the mean pollution detection time. We also find that the monitoring sensor’s pollution detection threshold also has little effect on the design of the optimum water quality monitoring network if it is smaller than the maximal pollutant concentration of a pollution event. However, the sensor’s pollution detection threshold will evidently affect the monitoring network design when it is larger than the maximal pollution concentration.

In this paper, we have mainly focused on theoretical-mathematical methods to design a multi-objective optimization algorithm for bidirectional rivers and verify its correctness and global optimization capability based on varies of simulations and experiments. A real river system can be indeed much more complex than the hypothetical river network. However, the use of SWMM would not affect the validation of our MOPSO algorithm. This is because our algorithm only accepts the simulation results of pollution detection time and potential monitoring locations to calculate optimal solutions. In fact, we have also used Qual2K to simulation pollution events based on the same hypothetical river network and got the same optimal deployment solutions. When we apply our algorithm to real river systems, we can use powerful business hydraulic simulation software (e.g., FLUENT, MIKE and InfoWorks) to simulate complex hydraulic situations (bed processes, dam, wetlands, simultaneous pollution events, different slops and widths, etc.) and obtain more accurate pollution detection times. Our algorithm can get better optimization results with more accurate hydraulic simulation results. The selection of sensors is also important for a real water quality monitoring network. We select special sensors based on various factors such as the type of pollutants we want to monitor, the pollution detection threshold we need, and the budget for building the monitoring system.

This novel algorithm will be applied to a real water quality monitoring network when we collect the necessary data. Further research is planned to explore the feasibility of integrating priority coefficients into MOPSO to guide the convergence processing. Finally, it is desirable to redesign the velocity and position functions with a fully discrete method to improve the computing performance.

## Figures and Tables

**Table 1:** Hydraulic characteristics of river network A.

Catchment	Width (ft)	Channel Slope	Manning’s Coefficient	Length (ft)	Flow Rate (ft^3^/s)
A	10	0.0001	0.02	2000	10
B	10	0.0001	0.02	2000	10
C	10	0.0001	0.02	2000	10
D	10	0.0001	0.02	2000	10
E	10	0.0001	0.02	1000	10
F	10	0.0001	0.02	2000	10
G	10	0.0001	0.02	3000	20
H	10	0.0001	0.02	4000	20
I	10	0.0001	0.02	2000	30
J	10	0.0001	0.02	3000	30
K	10	0.0001	0.02	5000	60

**Table 2:** Pollution detection time for river network A with a detection threshold of 0.01 mg/L.

Pollution Locations	Pollution Detection Time for Potential Monitoring Locations											
	1	2	3	4	5	6	7	8	9	10	11	12
1	0	27	-	81	-	118	-	-	-	-	-	198
2	-	0	-	40	-	75	-	-	-	-	-	152
3	-	27	0	81	-	118	-	-	-	-	-	198
4	-	-	-	0	-	23	-	-	-	-	-	96
5	-	-	-	28	0	62	-	-	-	-	-	139
6	-	-	-	-	-	0	-	-	-	-	-	62
7	-	-	-	-	-	38	0	-	-	-	-	113
8	-	-	-	-	-	79	27	0	-	-	-	157
9	-	-	-	-	-	111	57	-	0	-	-	190
10	-	-	-	-	-	133	78	-	10	0	-	213
11	-	-	-	-	-	156	99	-	27	-	0	236
12	-	-	-	-	-	-	-	-	-	-	-	0

**Table 3:** Optimal deployment solutions on Pareto frontier for river network A with a detection threshold of 0.01 mg/L.

Monitoring Locations	Detection Time (min)	Detection Probability
6, 9, 12	45.8	100%
2, 6, 9	26.6	91.7%
2, 7, 9	14.8	66.7%
2, 8, 9	13	58.3%
3, 7, 9	10.7	50.0%
5, 7, 9	10.7	50.0%
5, 8, 9	7.4	41.7%
3, 8, 9	7.4	41.7%
5, 9, 11	2.5	33.3%
5, 8, 11	0.0	25%
1, 5, 10	0.0	25%
5, 8, 10	0.0	25%
1, 5, 8	0.0	25%

**Table 4:** Pollution detection time for river network B with a detection threshold of 0.01 mg/L.

Pollution Locations	Pollution Detection Time for Potential Monitoring Locations											
	1	2	3	4	5	6	7	8	9	10	11	12
1	0	-	-	-	-	-	-	-	-	-	-	-
2	27	0	27	-	-	-	-	-	-	-	-	-
3	-	-	0	-	-	-	-	-	-	-	-	-
4	81	40	81	0	28	-	-	-	-	-	-	-
5	-	-	-	-	0	-	-	-	-	-	-	-
6	118	75	118	23	62	0	38	79	111	133	156	-
7	-	-	-	-	-	-	0	27	57	78	99	-
8	-	-	-	-	-	-	-	0	-	-	-	-
9	-	-	-	-	-	-	-	-	0	10	27	-
10	-	-	-	-	-	-	-	-	-	0	-	-
11	-	-	-	-	-	-	-	-	-	-	0	-
12	198	152	198	96	139	62	113	157	190	213	236	0

**Table 5:** Optimal deployment solutions on Pareto frontier for river network B with a detection threshold of 0.01 mg/L.

Monitoring Locations	Detection Time (min)	Detection Probability
3, 5, 10	38.2	75%
3, 4, 10	29.3	66.7%
4, 8, 10	22.3	58.3%
6, 8, 10	16.5	50.0%
4, 8, 12	10.0	41.7%
4, 5, 12	5.8	33.3%
4, 7, 12	5.8	33.3%
6, 7, 12	0.0	25%
4, 6, 12	0.0	25%

**Table 6:** Optimal deployment solutions for bidirectional water flows with a detection threshold of 0.01 mg/L.

Monitoring Locations	Detection Time (min)	Detection Probability
3, 10, 12	57.9	66.7%
3, 6, 10	31.6	58.3%
3, 6, 8	22.6	50.0%
5, 6, 8	11.6	41.7%
5, 6, 7	6.4	33.3%
4, 7, 8	0	25%
4, 8, 10	0	25%
3, 7, 9	0	25%
3, 9, 11	0	25%
6, 7, 9	0	25%

**Table 7:** Optimal deployment solutions for bidirectional water flows with a detection threshold of 0.01 mg/L.

**(a) Probability Ratio of River Networks A and B Is 70:30**		
**Monitoring Locations**	**Detection Time (min)**	**Detection Probability**
3, 10, 12	65.4	66.67%
3, 6, 10	33.1	58.33%
3, 6, 8	22.6	50.0%
5, 6, 8	11.8	41.67%
5, 7, 10	6.1	33.33%
4, 7, 8	0	25%
4, 8, 10	0	25%
3, 7, 9	0	25%
3, 9, 11	0	25%
6, 7, 9	0	25%
(**b**) **Probability Ratio of River Networks A and B Is 30:70**		
**Monitoring Locations**	**Detection Time (min)**	**Detection Probability**
3, 10, 12	50.5	66.67%
3, 6, 10	30.2	58.33%
3, 6, 8	22.1	50.0%
5, 6, 8	11.4	41.67%
5, 7, 10	6.0	33.33%
4, 7, 8	0	25%
4, 8, 10	0	25%
3, 7, 9	0	25%
3, 9, 11	0	25%
6, 7, 9	0	25%

**Table 8:** Pollution detection time for river network A with higher pollution detection thresholds.

**(a) Pollution Detection Threshold = 1 mg/L**												
**Pollution Locations**	**Pollution Detection Time for Potential Monitoring Locations**											
	**1**	**2**	**3**	**4**	**5**	**6**	**7**	**8**	**9**	**10**	**11**	**12**
1	0	44	-	112	-	165	-	-	-	-	-	253
2	-	0	-	61	-	110	-	-	-	-	-	199
3	-	44	0	112	-	165	-	-	-	-	-	253
4	-	-	-	0	-	42	-	-	-	-	-	131
5	-	-	-	47	0	97	-	-	-	-	-	186
6	-	-	-	-	-	0	-	-	-	-	-	90
7	-	-	-	-	-	62	0	-	-	-	-	152
8	-	-	-	-	-	116	47	0	-	-	-	205
9	-	-	-	-	-	153	82	-	0	-	-	242
10	-	-	-	-	-	181	108	-	20	0	-	269
11	-	-	-	-	-	208	134	-	44	-	0	297
12	-	-	-	-	-	-	-	-	-	-	-	0
**(b) Pollution Detection Threshold = 2 mg/L**												
**Pollution Locations**	**Pollution Detection Time for Potential Monitoring Locations**											
	**1**	**2**	**3**	**4**	**5**	**6**	**7**	**8**	**9**	**10**	**11**	**12**
1	0	50	-	124	-	-	-	-	-	-	-	-
2	-	0	-	69	-	-	-	-	-	-	-	-
3	-	50	0	124	-	-	-	-	-	-	-	-
4	-	-	-	0	-	-	-	-	-	-	-	-
5	-	-	-	55	0	-	-	-	-	-	-	-
6	-	-	-	-	-	-	-	-	-	-	-	-
7	-	-	-	-	-	-	0	-	-	-	-	-
8	-	-	-	-	-	-	55	0	-	-	-	-
9	-	-	-	-	-	-	92	-	0	-	-	-
10	-	-	-	-	-	-	119	-	24	0	-	-
11	-	-	-	-	-	-	146	-	50	-	0	-
12	-	-	-	-	-	-	-	-	-	-	-	-

**Table 9:** Pollution detection time for river network B with higher pollution detection thresholds.

**(a) Pollution Detection Threshold = 1 mg/L**												
**Pollution Locations**	**Pollution Detection Time for Potential Monitoring Locations**											
	**1**	**2**	**3**	**4**	**5**	**6**	**7**	**8**	**9**	**10**	**11**	**12**
1	0	-	-	-	-	-	-	-	-	-	-	-
2	44	0	44	-	-	-	-	-	-	-	-	-
3	-	-	0	-	-	-	-	-	-	-	-	-
4	112	61	112	0	47	-	-	-	-	-	-	-
5	-	-	-	-	0	-	-	-	-	-	-	-
6	165	110	165	42	97	0	62	116	153	181	208	-
7	-	-	-	-	-	-	0	47	82	108	134	-
8	-	-	-	-	-	-	-	0	-	-	-	-
9	-	-	-	-	-	-	-	-	0	20	44	-
10	-	-	-	-	-	-	-	-	-	0	-	-
11	-	-	-	-	-	-	-	-	-	-	0	-
12	253	199	253	131	186	90	152	205	242	269	297	0
**(b) Pollution Detection Threshold = 2 mg/L**												
**Pollution Locations**	**Pollution Detection Time for Potential Monitoring Locations**											
	**1**	**2**	**3**	**4**	**5**	**6**	**7**	**8**	**9**	**10**	**11**	**12**
1	0	-	-	-	-	-	-	-	-	-	-	-
2	50	0	50	-	-	-	-	-	-	-	-	-
3	-	-	0	-	-	-	-	-	-	-	-	-
4	124	69	124	0	55	-	-	-	-	-	-	-
5	-	-	-	-	0	-	-	-	-	-	-	-
6	-	-	-	-	-	-	-	-	-	-	-	-
7	-	-	-	-	-	-	0	55	92	119	146	-
8	-	-	-	-	-	-	-	0	-	-	-	-
9	-	-	-	-	-	-	-	-	0	24	50	-
10	-	-	-	-	-	-	-	-	-	0	-	-
11	-	-	-	-	-	-	-	-	-	-	0	-
12	-	-	-	-	-	-	-	-	-	-	-	-

**Table 10:** Optimal deployment solutions on Pareto frontier for bidirectional water flows.

**(a) Pollution Detection Threshold = 1 mg/L**		
**Monitoring Locations**	**Detection Time (min)**	**Detection Probability**
3, 10, 12	78.94	66.67%
3, 6, 10	46.5	58.33%
3, 6, 8	34.75	50.0%
5, 6, 8	19.8	41.67%
5, 7, 10	11.25	33.33%
4, 7, 8	0	25%
4, 8, 10	0	25%
3, 7, 9	0	25%
3, 9, 11	0	25%
6, 7, 9	0	25%
**(b) Pollution Detection Threshold = 2 mg/L**		
**Monitoring Locations**	**Detection Time (min)**	**Detection Probability**
5, 7, 10	14.5	33.3%
4, 7, 10	14.5	33.3%
3, 7, 10	14.5	33.3%
3, 4, 9	14.5	33.3%
3, 4, 7	14.5	33.3%
4, 7, 9	0	25%
4, 8, 10	0	25%
3, 7, 9	0	25%
3, 9, 11	0	25%
5, 7, 9	0	25%

## References

[1] Behmel S., Damour M., Ludwig R., Rodriguez M.J. (2016). Water quality monitoring strategies—A review and future perspectives. Sci. Total Environ..

[2] Park S.Y., Choi J.H., Wang S., Soon Park S. (2006). Design of a water quality monitoring network in a large river system using the genetic algorithm. Ecol. Model..

[3] Chilundo M., Kelderman P. (2008). Design of a water quality monitoring network for the Limpopo River Basin in Mozambique. Phys. Chem. Earth Parts A/B/C.

[4] Chang C.L., Lin Y.T. (2014). A water quality monitoring network design using fuzzy theory and multiple criteria analysis. Environ. Monit. Assess..

[5] Kim S.H., Aral M.M., Eun Y., Park J.J., Park C. (2017). Impact of sensor measurement error on sensor positioning in water quality monitoring networks. Stoch. Environ. Res. Risk Assess..

[6] Lee C., Paik K., Yoo D.G., Kim J.H. (2014). Efficient method for optimal placing of water quality monitoring stations for an ungauged basin. J. Environ. Manag..

[7] Tanos P., Kovács J., Kovács S., Anda A., Hatvani I.G. (2015). Optimization of the monitoring network on the River Tisza (Central Europe, Hungary) using combined cluster and discriminant analysis, taking seasonality into account. Environ. Monit. Assess..

[8] Chen Q., Wu W., Blanckaert K., Ma J., Huang G. (2012). Optimization of water quality monitoring network in a large river by combining measurements, a numerical model and matter-element analyses. J. Environ. Manag..

[9] Guestrin C., Krause A., Singh A.P. Near-optimal sensor placements in gaussian processes. Proceedings of the 22nd International Conference on Machine Learning.

[10] Strobl R.O., Robillard P.D. (2008). Network design for water quality monitoring of surface freshwaters: A review. J. Environ. Manag..

[11] Telci I.T., Nam K., Guan J., Aral M.M. (2009). Optimal water quality monitoring network design for river systems. J. Environ. Manag..

[12] Park C. (2013). Discrete Optimization via Simulation with Stochastic Constraints. Ph.D. Thesis.

[13] Aboutalebi M., Bozorg-Haddad O., Loáiciga H.A. (2016). Multiobjective Design of Water-Quality Monitoring Networks in River-Reservoir Systems. J. Environ. Eng..

[14] Rossman L.A. (2010). Storm Water Management Model User’s Manual.

[15] Ouyang H.T., Yu H., Lu C.H., Luo Y.H. (2008). Design optimization of river sampling network using genetic algorithms. J. Water Resour. Plan. Manag..

[16] Telci I.T., Nam K., Guan J., Aral M.M. (2008). Real time optimal monitoring network design in river networks. World Environmental and Water Resources Congress 2008: Ahupua’a.

[17] Reyes-Sierra M., Coello C.C. (2006). Multi-objective particle swarm optimizers: A survey of the state-of-the-art. Int. J. Comput. Intell. Res..

[18] Coello C.A., Lechuga M. MOPSO: A proposal for multiple objective particle swarm optimization. Proceedings of the 2002 Congress on Evolutionary Computation—2002, (CEC’02).

[19] Coello C.A.C., Pulido G.T., Lechuga M.S. (2004). Handling multiple objectives with particle swarm optimization. IEEE Trans. Evol. Comput..

